# The evolution of minimal residual disease: key insights based on a bibliometric visualization analysis from 2002 to 2022

**DOI:** 10.3389/fonc.2023.1186198

**Published:** 2023-07-18

**Authors:** Zhengyu Yu, Li Xie, Jing Zhang, Hua Lin, Ting Niu

**Affiliations:** ^1^ Department of Hematology, West China Hospital, Sichuan University, Chengdu, China; ^2^ State Key Laboratory of Wildlife Quarantine and Surveillance (Sichuan), Technology Center of Chengdu Customs, Chengdu, China

**Keywords:** minimal residual disease (MRD), bibliometrics, VOSviewer, CiteSpace, hematological malignancies

## Abstract

**Background:**

The topic of minimal residual disease (MRD) has emerged as a crucial subject matter in the domain of oncology in recent years. The detection and monitoring of MRD have become essential for the diagnosis, treatment, and prognosis of various types of malignancy.

**Aims:**

The purpose of this study is to explore the research trends, hotspots, and frontiers of MRD in the last two decades through bibliometric analysis.

**Methods:**

We employed Web of Science databases to carry out a bibliometric visualization analysis of research on 8,913 academic papers about MRD research from 2002 to 2022. VOSviewer, CiteSpace, RStudio, and a bibliometric online analysis platform were mainly used to conduct co-occurrence analysis and cooperative relationship analysis of countries/regions, institutions, journals, and authors in the literature. Furthermore, co-occurrence, co-citation, and burst analyses of keyword and reference were also conducted to generate relevant knowledge maps.

**Results:**

In the past 20 years, the number of MRD research papers has presented an overall rising trend, going through three stages: a plateau, development, and an explosion. The output of articles in the United States was notably superior and plays a dominant role in this field, and the Netherlands had the highest average citation per article. The most productive and influential institution was the University of Texas MD Anderson Cancer Center. *Blood* published the most papers and was the most cited journal. A collection of leading academics has come to the fore in the research field, the most prolific of which is Kantarjian HM. It was found that the application of MRD in “acute myeloid leukemia”, “acute lymphoblastic leukemia”, “multiple myeloma”, as well as the detection technology of MRD, are the research hotspots and frontiers in this domain. Furthermore, we analyzed the co-citation network of references and found that the top 10 co-cited references were all associated with MRD in hematological malignancies.

**Conclusion:**

This bibliometric visualization analysis conducted a thorough exploration into the research hotspots and trends in MRD from 2002 to 2022. Our findings can aid researchers in recognizing possible collaborations, guiding future research directions, and fostering the growth of MRD detection and monitoring technologies.

## 1 Introduction

Minimal residual disease (MRD) denotes the existence of malignant cells that remain in the body of a cancer patient following a particular treatment regimen, without clinical symptoms or signs of disease, and is not detectable by cellular morphology or routine screening methods. This elucidates the reasons that the majority of patients who attain a state of the complete response still relapse within years (1–3). MRD mainly applies to hematological malignancies and solid tumors (4). The detection and monitoring of MRD are essential in evaluating patient conditions, predicting recurrence risk, and formulating treatment plans to improve outcomes (5–7).

Detection of MRD primarily relies on real-time quantitative polymerase chain reaction (RQ-PCR), multiparametric flow cytometry (MFC), and next-generation sequencing (NGS). Among these methods, RQ-PCR is a relatively sensitive technique, and its monitoring targets include fusion genes (FGs), overexpressed genes, and gene mutations (8), with measured levels ranging from 10^-3^ to 10^-6^ (9–11). However, due to the heterogeneity of target molecules, specific primers and probes need to be designed, which limits their wider use (12). MFC is a widely used approach to the detection of MRD, which utilizes antibodies attached to particular markers upon the exterior of cancer cells. Through the labelling of these cells with fluorescence, a laser beam of flow cytometry can be used for analysis and quantification. The advantage of this technique is its ability to analyze multiple markers simultaneously with a sensitivity range of 10^-1^ to 10^-5^, making it a valuable tool for identifying MRD in different types of cancer (13). The next-generation flow (NGF) developed by the EuroFlow consortium, which incorporates an eight-color dual-tube antibody panel assay, can analyze up to 10^7^ cells concurrently, with a sensitivity of 2×10^−6^. In addition, NGF specimens need to be tested immediately after collection and dilution, requiring a higher degree of professionalism (14). NGS is a newer method for detecting MRD that allows for the simultaneous analysis of thousands of genes. It has high sensitivity and specificity and can detect MRD in patients with various types of cancer. However, NGS is relatively expensive, requires specialized equipment and expertise and has a long equipment turnover cycle (15). The efficacy of these methods is reliant on the genetic makeup of the tumor, the timing of detection, and the sensitivity of the diagnostic tests. Hence, schemes that employ a combination of test methods are likely to yield the most effective assessment results (16, 17). The recent focus on detecting and monitoring MRD in various types of malignancy has prompted the emergence of new MRD detection techniques and approaches, expanding the research area in this field. MRD monitoring can be utilized as an independent prognostic factor, and it is being increasingly acknowledged that it constitutes a valuable endpoint for cancer treatment or clinical trials (18, 19).

Bibliometric analysis is a quantitative method for analysing the characteristics of published literature using statistical and mathematical tools. This method provides insight into developments and trends in a field of study. In this context, we conducted a comprehensive collation of MRD research from 2002 to 2022 and identified the development trend and research hotspots in this field. The literature was obtained from the Web of Science (WoS) database and analyzed by visualization map software. This study aims to better understand the current status of MRD research, present research frontiers, and explore potential future research directions. To provide a meaningful scientific reference for the research and development of MRD monitoring.

## 2 Materials and methods

### 2.1 Research methods

Bibliometrics is an analytical approach that applies mathematical and statistical methods to assess and illustrate published literature on a particular subject (20). This research method can obtain and analyze important information such as the details of publication authors, keywords, institutions, countries, and references. The results will help to understand the development trend of a scientific field, research focus, and researcher cooperation relationship (21). Furthermore, using computer technology to present results graphically and visually can help to uncover hidden relationships within the data and make the results more comprehensive (22).

### 2.2 Data source and search strategy

This study delimits the scope of analysis, employs a strict literature search strategy, and selects WoS as the data source. The WoS includes relevant material from a wide range of research fields and is a high-quality digital database widely accepted by researchers worldwide (23). It exceeds other databases in functionality and complexity, with historically greater coverage (24). The process for data retrieval and collection is shown in **Supplementary Figure S1**. Data in this study were acquired from the Science Citation Index Expanded (SCI-E) of the Web of Science Core Collection (WoSCC) on November 22, 2022. For this particular study, the retrieval strategy was to use “minimal residual disease” or “measurable residual disease” or “molecular residual disease” as the subject search term to guarantee the comprehensive and precise retrieval of data. The search time range was from January 1, 2002, to October 30, 2022. Taking into account the completeness of the publication information, the publication types were selected as articles and review articles, and the language utilized in literatures was confined to English for the purpose of facilitating analysis. Retrieved publications were exported as plain text files with “full record and cited references”, and these files were named “download.txt.”. At the same time, all documents were exported in the tab-delimited file (UTF-8) with “full record and cited references” to be uploaded to a bibliometric online analysis platform to analyze trends of publications in countries/regions, and international collaborations. The data were searched, downloaded, and analyzed by two independent researchers, and differences in analysis between both were resolved through discussion with the third researcher.

### 2.3 Statistical and visual analysis

Data visualization technology is a very important research method and means in bibliometrics. In this way, the dynamics of a subject can be explored, that is, the temporal mapping from its knowledge base to the frontiers of research. VOSviewer and CiteSpace are two frequently employed data visualization analysis software programs. Using VOSviewer, a large-scale bibliometric map can be constructed to reflect the importance of items such as authors, keywords, institutions, and the strength of relationships with adjacent items through label views, density views, cluster density views and scatter views (25). CiteSpace is a Java application for analysing and visualizing co-occurrence networks. It can divide a time interval into several time slices, from which individual co-citation networks can be obtained to highlight the main changes between adjacent time slices (26). Moreover, CiteSpace can detect and visualize trends and changes in science over time (27).

Therefore, VOSviewer version 1.6.18 and CiteSpace version 6.1.4 were used as the main research tools in this study. In addition, BiblioShiny, a software package running in RStudio, and a bibliometric online analysis platform (https://bibliometric.com/) were used as complementary methods. Our endeavor involved bibliometric analysis of academic research on MRD that has been published in the last 20 years utilizing the aforementioned tools. Including the publication trends, the countries/regions’ performance and collaboration, the institutions’ performance, and the core journals, as well as the authors, and to discern the evolution of the research subject matter and uncover the changes in research hotspots through keyword and reference analysis.

## 3 Results

### 3.1 Basic performance information and publication trends

**Figure 1:**
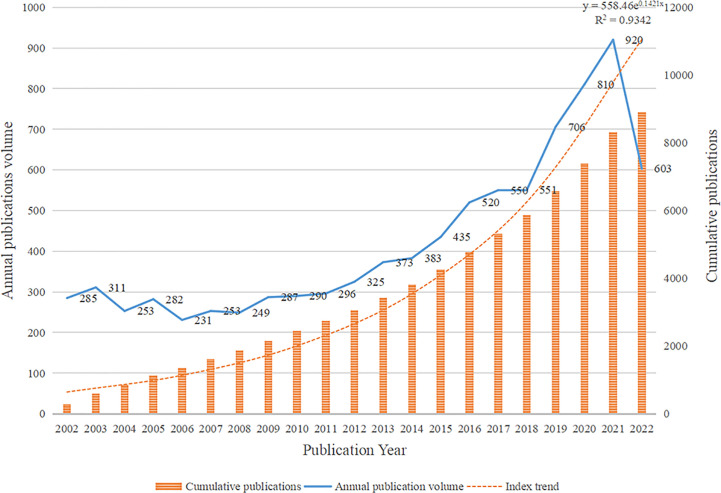
Distribution of publications on minimal residual disease (MRD) from 2002 to 2022.

A total of 8,913 papers, including 6,663 articles and 2,250 review articles, were included in this study. A total of 40,683 authors from 8,289 institutions in 86 countries/regions generated these articles, which were published in 996 journals and cited 175,916 references from 11,694 journals. The quantity and cumulative quantity of publications issued during distinct periods can serve as an indicator of the progression trajectory of this particular domain of inquiry. From January 1, 2002, to October 30, 2022, the temporal distribution of the identified 8,913 documents is illustrated in **Figure 1**. In the past two decades, the development trend of MRD-related publications can be broadly categorized into three phases. There was no significant change in the number of publications from 2002 to 2011, with an average of 273.7 articles published per year, indicating that the development of MRD-related research was slow during this period. From 2012 to 2018, the number of publications experienced a gradual increase, and research on MRD began to develop. The annual publication of relevant papers has shown explosive growth, especially after 2018, with an average annual increase of more than 100 papers for three consecutive years, indicating that MRD research has garnered increasing scholarly attention in recent years, leading to unparalleled development in the field. Linear regression analysis was carried out on the publication time of the literature and the annual cumulative number of publications, R^2 = ^0.9342. The model fitting is of high quality and consistent with Price’s scientific exponential growth law, which proposes that scientific metrics increase exponentially with time (28). The above results suggest that the relevant research on MRD is currently in a period of rapid development and that the speed of achievement output and literature publication is also accelerating.

### 3.2 Distribution of countries/regions

**Figure 2:**
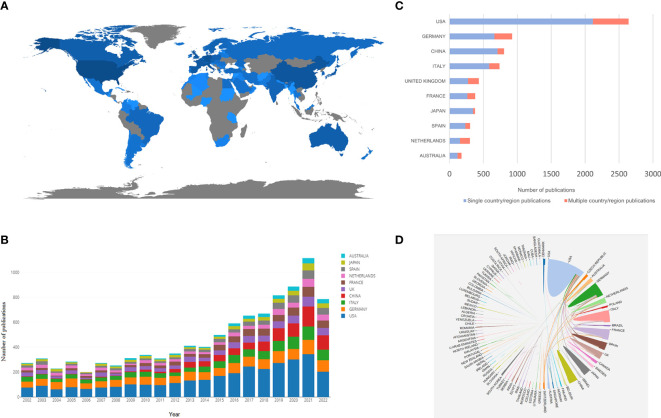
**(A)** The geographical distribution map based on the total publications of major countries/regions. **(B)** The changing trends in the number of publications in the top 10 countries/regions from 2002 to 2022. **(C)** The SCP and MCP in the top 10 corresponding author’s country/region. **(D)** Collaboration network between countries/regions related to MRD.

There were 86 countries/regions in MRD-relevant research in the past two decades. The geographical distribution of major countries/regions is shown in **Figure 2A**, with darker blue representing a greater number of publications. **Table 1** lists the top 10 countries/regions for MRD-related articles. The United States holds the top position in terms of the number of articles (3,190 papers, 179,255 total citations, 56.19 average citations per article), followed by Germany (1,456 papers, 104,112 total citations, 71.51 average citations per article) and Italy (1,078 papers, 67,837 total citations, 62.93 average citations per article). Although the total number of publications ranked fourth, China (915 papers, 18,619 total citations, 20.35 average citations per article) has made remarkable progress in the research output on MRD in recent years and surpassed Italy and Germany in 2018 and 2019, respectively, to become the second country in the number of publications (**Figure 2B**). Meanwhile, the Netherlands had the highest average citation per article, reaching 86.55. The top 10 countries/regions with the most publications are mostly from Europe and the United States, indicating that there are more achievements on MRD research in these regions. Based on the WoS, the H-index is a useful indicator to assess the impact and productivity of certain countries/regions, institutions, or scientists in an academic field (29). The H-index is a quantifiable metric of scholarly accomplishment that can be succinctly defined as the number of publications (h) that have garnered no less than h citations, with an H-index of 30 indicating that a scientist has 30 publications cited no less than 30 times. According to statistics, the top 3 countries/regions for the H-index are the United States (H-index = 185), Germany (H-index = 152), and the United Kingdom (H-index = 121).

**Table 1:** The top10 countries/regions with most publications related to minimal residual disease (MRD).

Runk	Countries/regions	Publications	Citations	Average Citations	H-index	Status of the Corresponding Author’s Country/region				
						Publications	Percentage	SCP	MCP	MCP Ratio
1	United States	3190	179255	56.19	185	2642	29.64	2114	528	19.98
2	Germany	1456	104112	71.51	152	925	10.38	663	262	28.32
3	Italy	1078	67837	62.93	114	738	8.28	585	153	20.73
4	China	915	18619	20.35	54	806	9.04	709	97	12.03
5	United Kingdom	813	63814	78.49	121	433	4.86	273	160	36.95
6	France	696	53098	76.29	101	379	4.25	264	115	30.34
7	Netherlands	572	49481	86.51	103	302	3.39	154	148	49.01
8	Spain	553	46974	84.94	98	303	3.40	234	69	22.77
9	Japan	455	24245	53.29	52	376	4.22	347	29	7.71
10	Australia	328	26211	79.91	66	177	1.99	119	58	32.77

SCP, single country/region publications; MCP, multiple country/region publications.

To further show the contribution of each country/region in the field of MRD research, we also counted the distribution of the corresponding author’s country/region. The United States remains the most productive country (2,642 papers, accounting for 29.64%) and outperforms other countries by a wide margin, followed by Germany (925 papers, accounting for 10.38%) and China (806 papers, accounting for 9.04%) (**Figure 2C**). Single country/region publications (SCP) stand for the number of publications co-authored by authors from the same country, whereas multiple country/region publications (MCP) stands for the number of papers co-authored with authors from multiple countries. According to the MCP ratio, the Netherlands, the United Kingdom, Australia, France, and Germany rank high in terms of international cooperation. However, although China and Japan achieved good performance in this field of research, they are not very active in participating in international cooperation, and their main partners are the United States (**Figure 2D**).

### 3.3 Contributions of institutions

**Figure 3:**
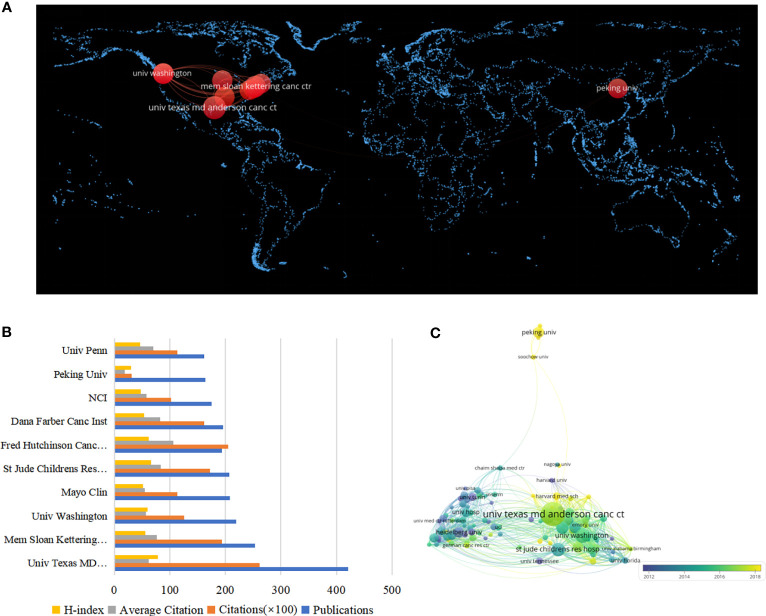
**(A)** The world distribution map of the top 10 institutions in the field of MRD. **(B)** The top ten institutions in the MRD field and their respective output performance. **(C)** The knowledge map of the institutions’ cooperation network related to MRD.

There were 8,289 institutions that contributed at least one article in this area of research. **Table 2** includes the top 10 institutions in terms of the number of publications in the field of MRD research. Among them, the University of Texas MD Anderson Cancer Center had the most published articles and the highest H-index value (421 papers, accounting for 4.72%, 62.04 average citations per article, H-index =78), followed by Memorial Sloan Kettering Cancer Center (253 papers, accounting for 2.84%, 76.39 average citations per article, H-index = 56) and University of Washington (219 papers, accounting for 2.46%, 57.22 average citations per article, H-index = 60). The average citation per article of Fred Hutchinson Cancer Center was the highest, reaching 105.82, suggesting that articles published by this institution are widely followed. In addition, Peking University, as the only non-US institution in the top 10, also had good performance (164 papers, accounting for 1.84%, 19.07 average citations per article, H-index = 30), but the average number of citations per article and the H-index were not high (**Figure 3B**). **Figure 3A** shows the world distribution of the top 10 institutions in the field of MRD, 90% of which are in the United States, indicating that scientific institutions in America have played a crucial role in advancing progress in this domain. These institutions have important academic prestige and authority in various fields. Among them, the University of Texas MD Anderson Cancer Center, Memorial Sloan Kettering Cancer Center, and Mayo Clinic are the top 3 best hospitals for cancer ranks by U.S. NEWS (https://health.usnews.com/best-hospitals/rankings/cancer). VOSviewer produces a network visualization map to show institutional collaboration. Each node represents one institution, 103 institutions are presented in **Figure 3C**, and at least 50 articles were published by them. The size of the nodes depicted in the figure are indicative of the number of articles published by the respective institutions, whereas the thickness of the connecting lines denotes the degree of collaboration in publishing between them. The institutions located in both Europe and the United States engage in frequent and close cooperation with each other.

**Table 2:** The top 10 institutions with most publications related to MRD.

Runk	Institutions	Publications	Percentage	Citations	Average Citations	H-index	Country
1	Univ Texas MD Anderson Canc Ctr	421	4.72	26118	62.04	78	United States
2	Mem Sloan Kettering Canc Ctr	253	2.84	19327	76.39	56	United States
3	Univ Washington	219	2.46	12531	57.22	60	United States
4	Mayo Clin	208	2.33	11370	54.66	52	United States
5	St Jude Childrens Res Hosp	207	2.32	17230	83.24	66	United States
6	Fred Hutchinson Canc Res Ctr	194	2.18	20529	105.82	62	United States
7	Dana Farber Canc Inst	196	2.20	16197	82.64	54	United States
8	NCI	175	1.96	10163	58.07	47	United States
9	Peking Univ	164	1.84	3127	19.07	30	China
10	Univ Penn	162	1.82	11304	69.78	46	United States

### 3.4 Journals and cited journals analysis

**Figure 4:**
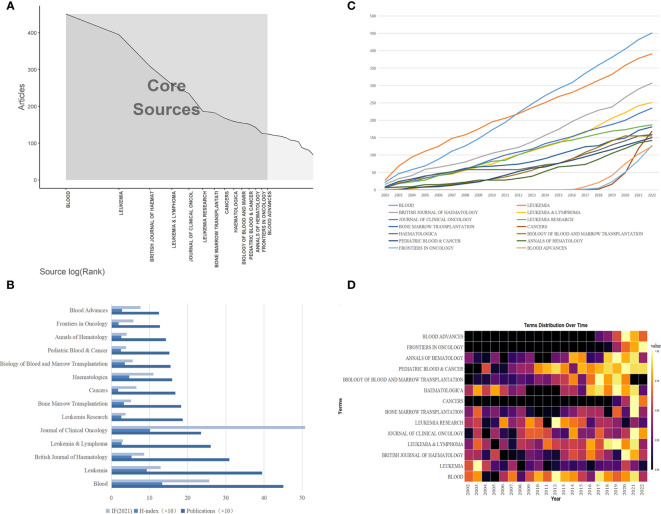
**(A)** Map of the distribution of core journals in the MRD field. **(B)** Core journals in the MRD field and their respective output performance and influence. **(C)** The dynamics of annual cumulative publications for core journals in the MRD field. **(D)** The heatmap of the annual publication dynamics of core journals in the MRD field. (Each box represents the yearly count of papers released by the journal, with greater brightness indicating a larger number of papers released).

A total of 996 journals published these 8,913 papers. Bradford’s Law states that if journals in a professional field are ranked in decreasing order by the number of papers published, the resulting list can be divided into core, related, and edge zones, each containing an equal number of papers. At this time, the number of core journals and other regional journals is proportional to 1: a: a^2^ (30). Therefore, using BiblioShiny, we found the core journals in the field of MRD research (**Figure 4A**), and a total of 3,049 articles were published in 14 core journals, accounting for 34.21% of all publications. *Blood* published the most papers and had the highest average number of citations per article (451 papers, accounting for 5.06%, 134.50 average citations per article, Q1), followed by *Leukemia* (395 papers, accounting for 4.43%, 79.49 average citations per article, Q1) and the *British Journal of Haematology* (309 papers, accounting for 3.47%, 29.55 average citations per article, Q1) (**Table 3**), which are the top journals in hematology and oncology. The 14 core journals are all published in Europe or the United States, with the United States and the United Kingdom publishing the most journals (n = 5), followed by Switzerland (n = 2), Germany and Italy (n = 1 for each). Among journals that published 125 or more articles, *Journal of Clinical Oncology* had the highest impact factor (IF 2021 = 50.739), followed by *Blood* (IF 2021 = 25.669) and *Leukemia* (IF 2021 = 12.897). At the same time, the H-index of these three journals is also in the top 3: *Blood* (H-index = 133), followed by *Journal of Clinical Oncology* (H-index = 101) and *Leukemia* (H-index = 93) (**Figure 4B**).

**Table 3:** The core journals related to MBD.

Runk	Journals	Publications	Percentage	Citations	Average Citations	H-index	IF(2021)	Category	Quartile in Category	Region
1	Blood	451	5.06	60659	134.50	133	25.669	Hematology	Q1	United States
2	Leukemia	395	4.43	31398	79.49	93	12.897	Hematology/Oncology	Q1/Q1	England
3	British Journal of Haematology	309	3.47	9132	29.55	53	8.615	Hematology	Q1	England
4	Leukemia & Lymphoma	260	2.92	3112	11.97	27	2.996	Hematology/Oncology	Q3/Q3	England
5	Journal of Clinical Oncology	235	2.64	30911	131.54	101	50.739	Oncology	Q1	United States
6	Leukemia Research	187	2.10	2775	14.84	26	3.715	Hematology/Oncology	Q3/Q3	England
7	Bone Marrow Transplantation	183	2.05	3721	20.33	33	5.174	Hematology/Immunology/Oncology/Transplantation	Q2/Q2/Q2/Q2	England
8	Cancers	168	1.88	1060	6.31	18	6.575	Oncology	Q1	Switzerland
9	Haematologica	159	1.78	6317	39.73	47	11.049	Hematology	Q1	Italy
10	Biology of Blood and Marrow Transplantation	155	1.74	4016	25.91	35	5.609	Hematology/Immunology/Transplantation	Q2/Q2/Q1	United States
11	Pediatric Blood & Cancer	152	1.71	2167	14.26	25	3.838	Hematology/Oncology/Pediatrics	Q2/Q3/Q1	United States
12	Annals of Hematology	143	1.60	1986	13.89	26	4.030	Hematology	Q2	Germany
13	Frontiers in Oncology	127	1.42	1076	8.47	19	5.738	Oncology	Q2	Switzerland
14	Blood Advances	125	1.40	2270	18.16	28	7.642	Hematology	Q1	United States

The dynamics of annual cumulative publications for these journals are shown in **Figure 4C**, while we mapped a heatmap of the annual publication dynamics of the journals using RStudio in **Figure 4D**. The number of articles related to MRD published in *Blood* has continued to increase over the years, and in 2012, it overtook *Leukemia* to become the journal with the most MRD-related articles. In particular, *Blood Advances* was founded in 2016 and has become a core journal for publishing MRD research in just a few years, with *Cancers* publishing 70 articles on MRD in 2021, making it the eighth position in terms of cumulative publications. The achievements of these journals are also remarkable.

**Figure 5:**
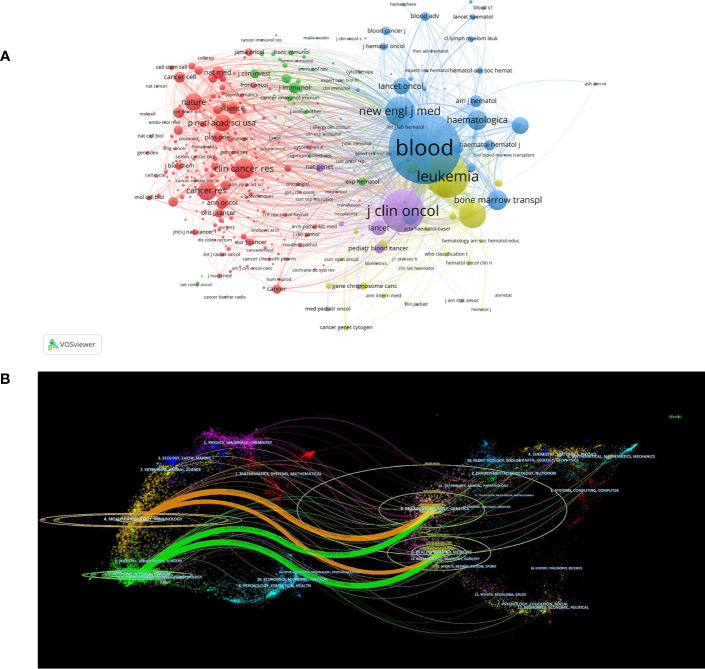
**(A)** The co-occurrence visualization map of co-cited journals. **(B)** The dual-map overlay of journals related to MRD research.

The notion of journal co-citation denotes the situation of two or more journals being cited by a single journal, thereby indicating the interconnection between the journals. **Supplementary Table S1** lists the top 10 co-cited journals related to MRD. *Blood* is the most co-cited journal (88,753 citations, IF 2021 = 25.669, H-index = 133, Q1), followed by *Leukemia* (34,699 citations, IF 2021 = 12.897, H-index = 93, Q1) and *Journal of Clinical Oncology* (33,963 citations, IF 2021 = 50.739, H-index = 101, Q1). The findings suggest that these three journals have had a great influence on MRD research and are receiving wide attention. The co-occurrence visualization map of co-cited journals was obtained by VOSviewer (**Figure 5A**). The node labels on the visualization map describe the 1000 journals cited by MRD studies, and the lines describe their co-citation relationships. The dual-map overlay of journals shows the position of a research subject relative to the main research science. Each dot on the map represents a journal, and the labels describe the various research areas covered by all journals. The journals that cite literature are positioned on the left side of the map, while those that are being cited are positioned on the right side. The colored curve is the citation line, showing the citation relationship, and the width of the curve is closely related to the citation frequency. The longer horizontal axis of the ellipse represents more papers published in the corresponding journal, and the longer vertical axis represents more authors (31). **Figure 5B** shows the four main citation paths, indicating that studies on MRD published in Molecular/Biology/Immunology journals and Medicine/Medical/Clinical journals citing studies in Molecular/Biology/Genetics and Health/Nursing/Medicine Journals.

### 3.5 Author and co-cited author analysis

To highlight the collaboration among the primary authors within this particular domain, authors with more than 27 publications are visualized in **Supplementary Figure S2A**, which consists of 102 nodes, representing 102 authors who have published 27 or more papers. The size of each node on the map corresponds to the number of articles authored by each individual, while the width of the interconnecting line signifies the degree of collaborative publishing among authors. Each color signifies a distinct cluster, and authors whose nodes are of the same color cooperate closely. We can see that some collaborative relationship were established among the highly productive authors, and stable research teams were formed.

The publications pertaining to MRD have been sourced from 40,683 authors, and the top 3 authors in terms of number of publications are Kantarjian HM (166 papers, accounting for 1.86%, 52.42 average citations per article), Huang XJ (130 papers, accounting for 1.46%, 20.64 average citations per article) and Schrappe M (117 papers, accounting for 1.31%, 79.44 average citations per article) (**Table 4**). A researchers’ academic productivity and quality can be evaluated through the use of the H-index, which is a blended quantitative indicator. The top 3 authors for the H-index are Kantarjian HM (H-index = 53), van Dongen JJM (H-index = 53) and Schrappe M (H-index = 51). The Dutch scholar van Dongen JJM has the highest citation frequency per article and H-index. Among the top 10 most productive authors, 4 were from the United States, and 3 were from China. It shows that American authors have made exceptional contributions in this particular domain. Although the output of articles by Chinese authors is relatively high, the citation frequency of each article and the H-index are not too high, indicating that Chinese authors are important participants in the field of MRD research, but their international academic influence needs to be improved.

**Table 4:** The top 10 authors with most publications related to MRD.

Runk	Author	Publications	Percentage	Citations	Average Citations	H-index	Location
1	Kantarjian HM	166	1.86	8701	52.42	53	United States
2	Huang XJ	130	1.46	2683	20.64	27	China
3	Schrappe M	117	1.31	9294	79.44	51	Germany
4	Ravandi F	116	1.30	4173	35.97	37	United States
5	Van dongen JJM	111	1.25	13962	125.78	53	Netherlands
6	Jabbour EJ	108	1.21	4199	38.88	37	United States
7	Orfao A	99	1.11	8260	83.43	44	Spain
8	Wang Y	99	1.11	1801	18.19	22	China
9	Xu LP	96	1.08	2193	22.84	25	China
10	O’brien S	89	1.00	5951	66.87	39	United States

In the past two decades of MRD research, there were 9,1867 cited authors. **Supplementary Figure S2B** shows a total of 101 authors cited more than or equal to 338 times. Co-cited authors refer to two or more authors who are cited by the same article, which can identify the high-impact research teams in this field (32). The American scholar Pui CH was the most frequently cited (2,062 citations, H-index = 48), followed by two Dutch scholars van Dongen JJM (1,525 citations, H-index = 53) and van der Velden VHJ (1,334 citations, H-index = 44). Moreover, Kantarjian HM (1,067 citations, H-index = 53) from the United States was also one of the highest H-index among the co-cited authors (**Supplementary Table S2**). It is proven that these four co-cited authors and their teams have a considerable contribution and great academic research prestige to the study of MRD.

### 3.6 Analysis of keywords

#### 3.6.1 High frequency and high centrality keyword analysis

The keywords represent the paper’s focus and reflect the paper’s disciplinary structure and research topics to a certain extent. Using the keyword co-occurrence network, the disciplinary structure and research focus of the analyzed object can be displayed clearly. CiteSpace was used to divide the analysis objects by one year per slice, the node type was selected as keyword, and the cosine algorithm was used to select the association strength of network nodes within slices. The threshold (top N) was set as 30, that is, the top 30 high-frequency keywords in each time slice were extracted. The pathfinding algorithm was chosen in this study, the pruning sliced network and pruning the merged network were used to show a clearer co-occurrence network.

**Figure 6:**
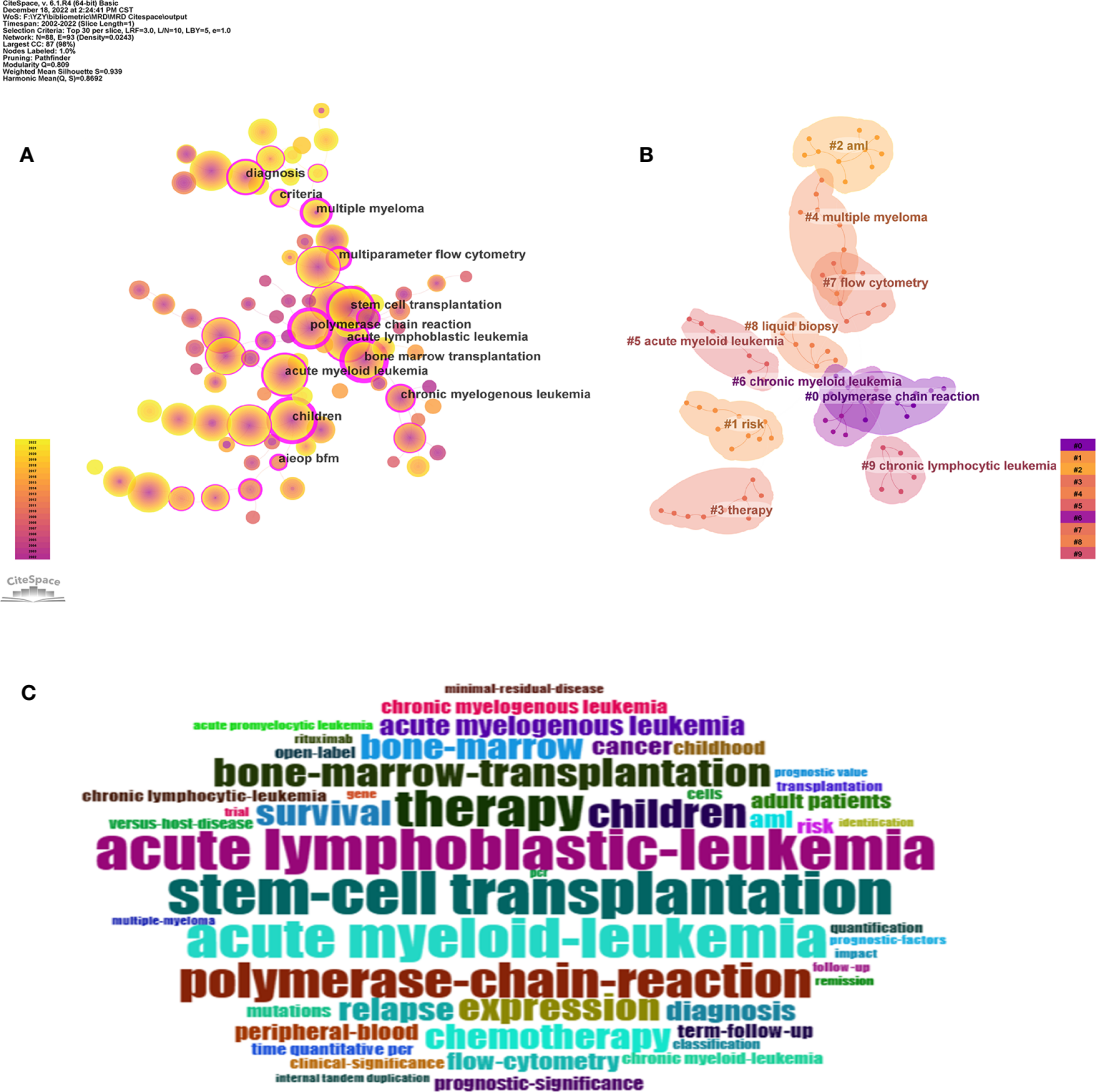
**(A)** The knowledge map of keyword co-occurrence network in the MRD research field. (The labels indicate high betweenness centrality keywords). **(B)** The knowledge map of keyword clustering network in the MRD research field. **(C)** The keyword cloud.

The keyword co-occurrence network in the MRD research field consisted of 88 nodes and 284 links, each node representing a keyword. The size of nodes and the frequency of keywords are positively correlated, signifying that larger nodes tend to have a greater frequency of keywords, and more purple around the node indicates a greater value of betweenness centrality. Correspondingly, the chromaticity of the node denotes the chronology: the hotter the tinge, the newer the epoch, and the cooler the tinge, the older the era (**Figure 6A**). **Table 5** outlines the top 20 keywords in frequency and betweenness centrality, providing the most prevalent and influential terms. High-frequency keywords are closely related to the research topic, and “stem cell transplantation”, “acute lymphoblastic leukemia”, “acute myeloid leukemia”, “therapy”, and “polymerase chain reaction” are keywords with a frequency of more than 900. Moreover, the more prominent the betweenness centrality of nodes in the network, the more significant the influence of the keywords they embody within the domain. The top 5 keywords of highly betweenness centrality are “bone marrow transplantation” (0.92), “children” (0.71), “polymerase chain reaction” (0.68), “stem cell transplantation” (0.6), and “multiparameter flow cytometry” (0.57).

**Table 5:** The top20 keywords for frequency and betweenness centrality related to MRD.

Runk	Keywords	Frequency	Runk	Keywords	Centrality
1	stem cell transplantation	1112	1	bone marrow transplantation	0.92
2	acute lymphoblastic leukemia	1078	2	children	0.71
3	acute myeloid leukemia	1036	3	polymerase chain reaction	0.68
4	therapy	957	4	stem cell transplantation	0.6
5	polymerase chain reaction	903	5	multiparameter flow cytometry	0.57
6	children	737	6	multiple myeloma	0.56
7	bone marrow transplantation	734	7	criteria	0.37
8	expression	710	8	diagnosis	0.34
9	survival	662	9	AIEOP-BFM	0.34
10	chemotherapy	661	10	acute lymphoblastic leukemia	0.3
11	bone-marrow	651	11	acute myeloid leukemia	0.3
12	relapse	593	12	chronic myelogenous leukemia	0.3
13	diagnosis	514	13	clinical significance	0.26
14	acute myelogenous leukemia	485	14	non-Hodgkin’s lymphoma	0.22
15	cancer	450	15	RT-PCR	0.22
16	AML	449	16	flow cytometry	0.18
17	flow cytometry	445	17	relapse	0.18
18	peripheral blood	424	18	AML	0.18
19	adult patients	394	19	prognostic factor	0.18
20	risk	371	20	internal tandem duplication	0.18

Through keyword analysis, this study obtained the keywords of hematological malignancies, such as “acute lymphoblastic leukemia”, “acute myeloid leukemia”, “multiple myeloma”, “chronic myelogenous leukemia”, and “non-Hodgkin’s lymphoma”. The keywords that reflected the treatment of hematological diseases, such as “stem cell transplantation”, “bone marrow transplantation”, “therapy”, “chemotherapy”, etc. The keywords that reflected techniques to measure MRD, such as “polymerase chain reaction”, “flow cytometry”, “multiparameter flow cytometry”, etc. Then, there were some keywords closely related to MRD, such as “relapse”, “survival”, “diagnosis”, “clinical significance”, and “prognostic factor”. In addition, to verify the reliability of the core keywords, BiblioShiny was also used to generate the keyword cloud (**Figure 6C**), revealing that the core keywords are roughly the same as those summarized above.

#### 3.6.2 keyword cluster analysis

Keyword clustering forms small groups of closely related keywords to realize the purpose of mining hidden information. It probed certain themes that have been established or may have been overlooked in a particular area of study. In this study, CiteSpace was used to perform keyword clustering analysis based on keyword co-occurrence, using the log-likelihood ratio (LLR) algorithm to perform cluster labels extracted from the publication keywords, as shown in **Figure 6B**. In general, there are two important metrics to evaluate the effect of cluster formation. The modularity Q is a metric utilized for evaluating the efficacy of network modularity, and a higher value indicates a better cluster obtained by the network. If Q>0.3, it indicates that the cluster structure is statistically significant. The homogeneity of cluster members is evaluated using the cluster silhouette value S, where S>0.5 implies high consistency among cluster members and a reasonable clustering outcome, and S>0.7 suggests high credibility of the result. The Q value of the cluster formation was 0.809, and the S value was 0.939, indicating that the clustering structure is significant, and the clustering results are convincing. Under the exploration of this study, 10 meaningful clusters were formed (**Supplementary Table S3**). The clusters are sorted from #0 to #9 in decreasing order of the number of keywords they comprise, with cluster #0 containing the most keywords.

#### 3.6.3 Analysis of keyword bursts

**Figure 7:**
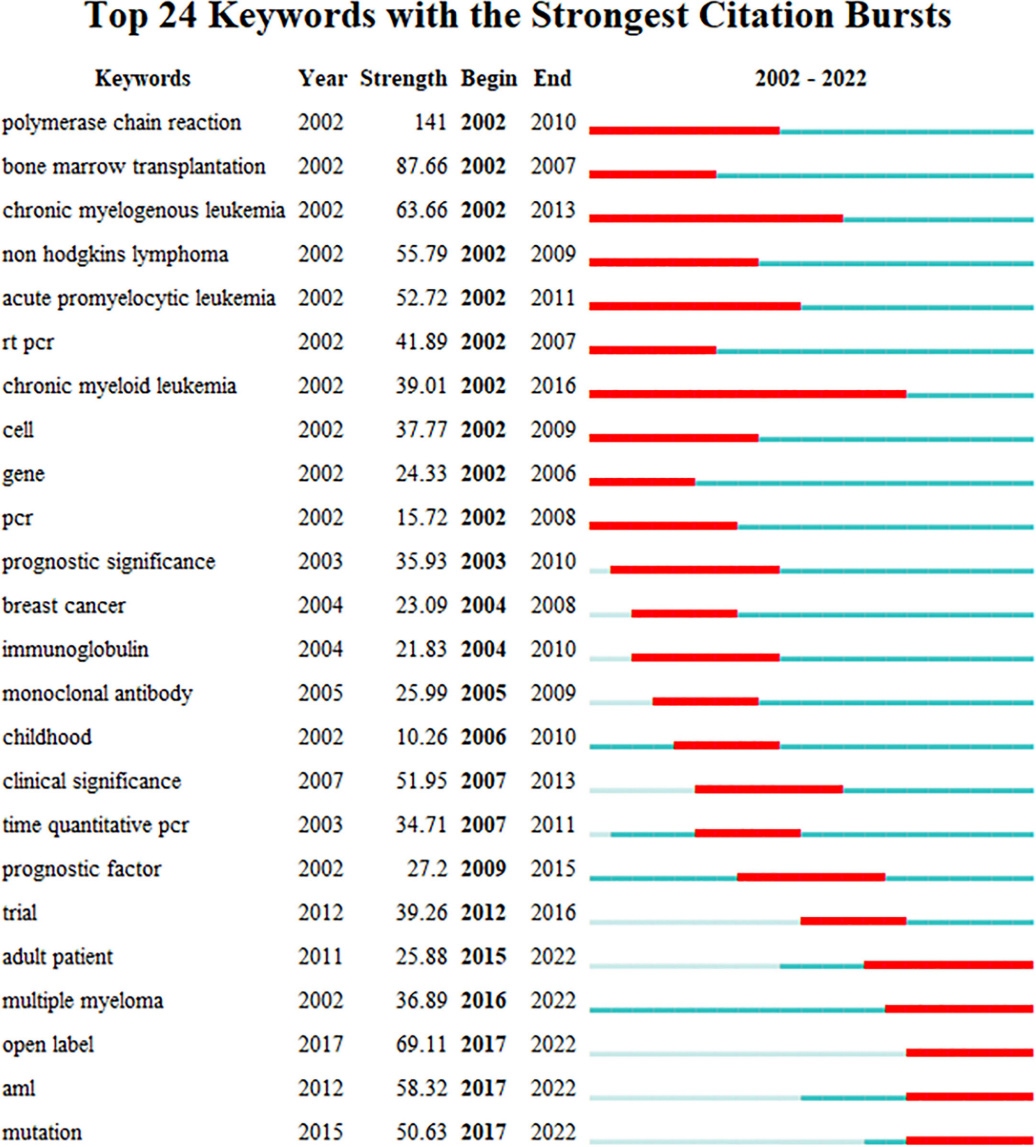
The top 24 keywords with the strongest citation bursts from 2002 to 2022.

As a refined expression of research topics and content in academic papers, keywords can reflect the research hotspots in the subject area to a certain extent. Keyword burst detection can be used to explore the sudden increase in research interest in a subject. The information can not only elucidate the dynamics of research hotspots over time but also expose research trends in recent years (22). Through the imposition of a minimum burst duration of 5 years, the identification of the top 24 keywords exhibiting the strongest citation bursts was accomplished, as depicted in **Figure 7**. The blue line indicates the year from the beginning to the end of the keyword, and the red line indicates the period when the keyword burst. The stronger the burst strength of the keyword, the more studies related to it. Among the top 24 keywords with the strongest citation bursts, “polymerase chain reaction” was the keyword with the greatest burst intensity (strength = 141), and a large number of research results were related to this word between 2002 and 2010. At the same time, “chronic myeloid leukemia” is the hot keyword with the longest duration (2002 to 2016). Furthermore, the citation bursts of 5 keywords continued through 2022. Therefore, the 5 burst keywords of “adult patient”, “multiple myeloma”, “open label”, “aml”, and “mutation” reflect the latest research hotspots in the field of MRD.

In general, in the early period of 2002-2022, the research hotspots of MRD mainly focused on chronic myeloid leukemia (CML), non-Hodgkin’s lymphoma (NHL), and chronic prolymphocytic leukemia. The technical methods mainly focused on the relationship between the results of gene-related polymerase chain reaction (PCR) detection and the prognosis of the disease. In the later period, MRD in acute myeloid leukemia (AML) and multiple myeloma (MM) attracted much attention, and gene mutations have become the research upsurge of MRD detection. In addition, the subjects of MRD also tended to develop from children to adults.

### 3.7 Analysis of co-cited references and reference burst

**Figure 8:**
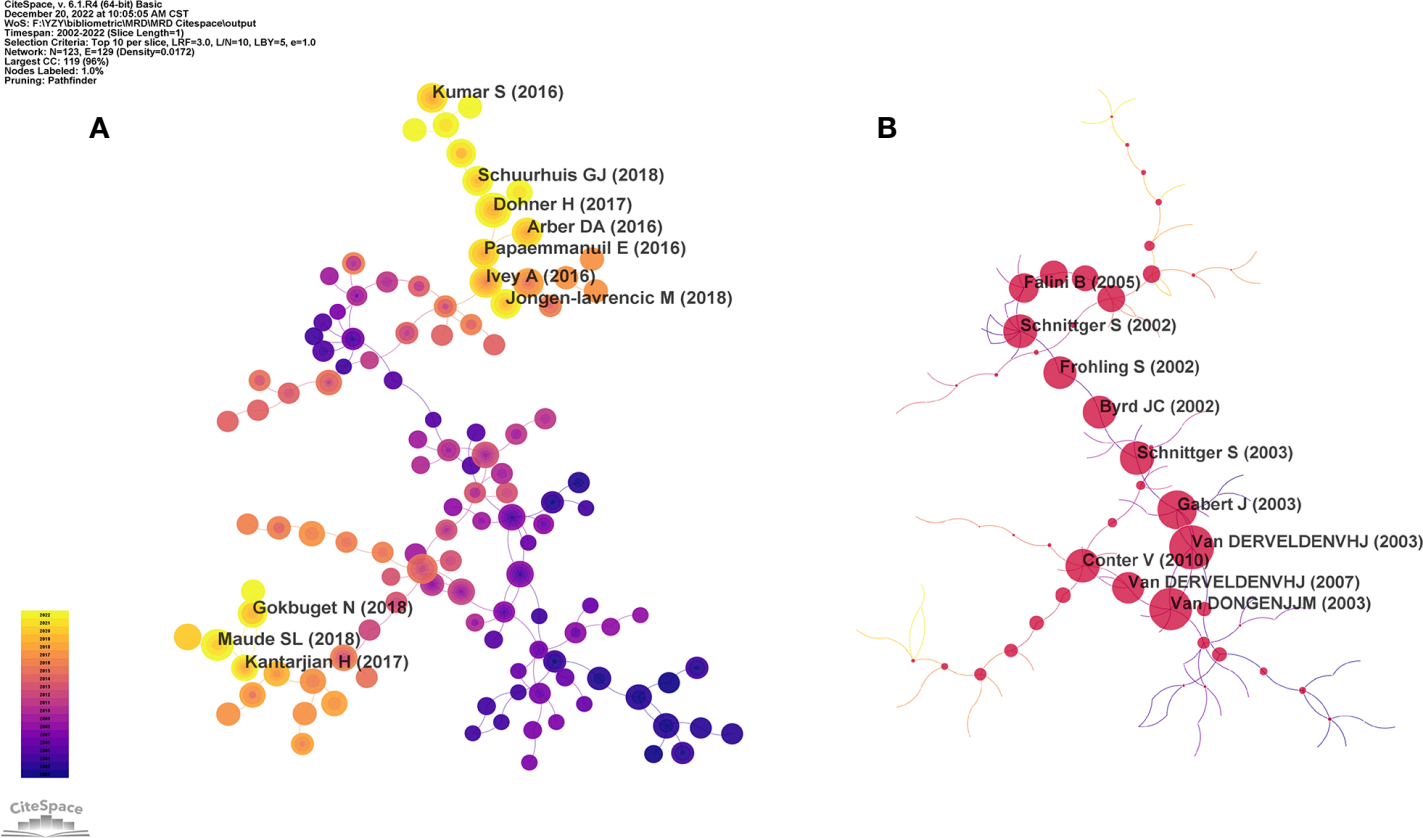
**(A)** The knowledge map of high frequency co-cited references network related to MRD. **(B)** The knowledge map of high betweenness centrality co-cited references network related to MRD.

A comprehensive knowledge system will invariably underpin any research topic. As a research topic matures, its knowledge system becomes completer and more enriched, largely due to the knowledge flow from the references. The examination of a research topic’s co-citation network can offer valuable insight into its knowledge base, with high-frequency and high betweenness centrality nodes being key points of focus. In addition, citation analysis can be used to evaluate the scientific value and influence of publications in a particular field of research and has a significant impact on the discussion, practice, and future research in this area (33). A total of 175,916 references were cited in the scope of our analysis. In CiteSpace, the threshold (top N) was set as 10, that is, the top 10 co-cited references in each time slice were extracted. In the past two decades, the network map of co-cited references related to MRD consists of 123 nodes and 194 links, and each node represents one reference, the lighter the node, the closer the time, and the darker the color, the more distant the time (**Figure 8A**). Interestingly, the top 10 highly cited references in this field were published between 2016 and 2018 and have received a high citation frequency, particularly in recent years. This suggests that research on MRD is progressing quickly, and the knowledge structure is being updated rapidly.

The high frequency nodes represent literature with a high citation rate, which is an important knowledge base in this field. The top 10 co-cited references were published in *New England Journal of Medicine* (IF 2021 = 176.082, n=5), *Blood* (IF 2021 = 25.669, n=4) and *Lancet Oncology* (IF 2021 = 54.433, n=1) from 2016 to 2018 (**Table 6**). These three journals are the top in related fields and have great academic influence. This indicates that the knowledge sources and basics in the field of MRD research are of high level and quality. Among them, the paper published in *Blood* by Dohner H in 2017 received the highest citation frequency. Based on the research progress of AML, Dohner H et al. published the 2017 edition of the European LeukemiaNet (ELN) recommendations for the diagnosis and management of AML in Adults, which revised the ELN gene categories, recommendations for response categories based on MRD status, and criteria for disease progression and have been widely adopted by doctors and scholars studying AML in clinical practice (33). The second most cited paper was published in *Blood* in 2018. To make the MRD of AML more standardized and instructive in clinical practice, a consensus document from the ELN has identified the key issues of MRD monitoring in AML. Examples include the prognostic threshold of molecular MRD markers in AML patients with morphological complete response, approaches for MFC MRD assessment, approaches for molecular MRD assessment, and thresholds and time points for MRD assessment during treatment/follow-up/relapse definition. The clinical application guidelines of MRD measurement in AML were also established (16). Ivey A et al. published the third most frequently co-cited study in *New England Journal of Medicine*. They performed MRD detection (as determined by quantification of the gene encoding nucleophosmin (NPM1)-mutated transcripts) in 2,569 samples from 346 patients with AML who had received intensive treatment. The results of the study showed that a marked association was established between the persistence of NPM1 mutated transcripts in the blood of patients after their second chemotherapy cycle and a greater probability of relapse and lower rates of survival at follow-up after three years. The NPM1 mutation is a reliable marker for detection in the majority of AML patients and is a powerful independent factor that predicts disease progression and prognosis. Until then, the measurement of MRD had been used only for acute promyelocytic leukemia and childhood acute lymphoblastic leukemia (ALL) (34).

**Table 6:** The top 10 co-cited references with the citation frequency related to MRD.

Runk	Co-cited reference	Citation frequency	First author (publication year)	Journal	IF(2021)	Quartile in category
1	Diagnosis and management of AML in adults: 2017 ELN recommendations from an international expert panel	492	Dohner H (2017)	Blood	25.669	Q1
2	Minimal/measurable residual disease in AML: a consensus document from the European LeukemiaNet MRD Working Party	327	Schuurhuis GJ (2018)	Blood	25.669	Q1
3	Assessment of Minimal Residual Disease in Standard-Risk AML	322	Ivey A (2016)	New England Journal of Medicine	176.082	Q1
4	International Myeloma Working Group consensus criteria for response and minimal residual disease assessment in multiple myeloma	259	Kumar S (2016)	Lancet Oncology	54.433	Q1
5	Blinatumomab versus Chemotherapy for Advanced Acute Lymphoblastic Leukemia	235	Kantarjian H (2017)	New England Journal of Medicine	176.082	Q1
6	Molecular Minimal Residual Disease in Acute Myeloid Leukemia	229	Jongen-lavrencic M (2018)	New England Journal of Medicine	176.082	Q1
7	The 2016 revision to the World Health Organization classification of myeloid neoplasms and acute leukemia	213	Arber DA (2016)	Blood	25.669	Q1
8	Genomic Classification and Prognosis in Acute Myeloid Leukemia	203	Papaemmanuil E (2016)	New England Journal of Medicine	176.082	Q1
9	Tisagenlecleucel in Children and Young Adults with B-Cell Lymphoblastic Leukemia	164	Maude SL (2018)	New England Journal of Medicine	176.082	Q1
10	Blinatumomab for minimal residual disease in adults with B-cell precursor acute lymphoblastic leukemia	147	Gokbuget N (2018)	Blood	25.669	Q1

A node with a high betweenness centrality is indicative of literature that is linked to multiple other literatures through a co-citation relationship and plays a pivotal role as a hub in the network. In **Figure 8B**, the red nodes represent co-cited references with high betweenness centrality, where the magnitude of the nodes is commensurate with their betweenness centrality value, signifying their importance in the overall knowledge network. These references are located at the key nodes of the co-citation map and run through the entire network. The top 10 co-cited references with the betweenness centrality related to MRD were published in *Blood* (IF 2021 = 25.669, n=5), *Leukemia* (IF 2021 = 12.897, n=4), and *New England Journal of Medicine* (IF 2021 = 176.082, n=1) (**Supplementary Table S4**). The top 3 references with betweenness centrality were all published in Leukemia in 2003. The ranked first was published by van der Velden VHJ et al. This review comprehensively summarized the application of RQ-PCR in the monitoring of MRD in hematological malignancies. Covering the principle of the RQ-PCR technique, the three main MRD-PCR target categories in hematological malignancies and their advantages and disadvantages, the sensitivity and specificity analysis of RQ-PCR, and the selection of control genes for MRD detection in hematological malignancies. The article emphasizes that while MRD monitoring guides treatment for some hematologic malignancy patients, RQ-PCR-based MRD detection requires further standardization and unification of data interpretation and laboratory reports in multicenter clinical treatment protocols and highlights the crucial role of regular quality control rounds for testing laboratories (35). Van Dongen JJM et al. published the second-ranked publication with betweenness centrality. Clonality analysis of gene rearrangement plays an important role in the diagnosis of lymphoproliferative lesions. However, the complexity and diversity of the immunoglobulin (Ig) and T-cell receptor (TCR) gene rearrangement process and the limitation of primer design result in a high rate of false negatives and false positives, thus restricting its usage. In 2003, the European BIOMED-2 collaboration group proposed a standardized PCR detection system for Ig/TCR gene rearrangement. This system included the design of multiple primers for the Ig/TCR locus, which were intended to cover a wide range of gene segments and almost all types of Ig/TCR gene rearrangements. As a result, 107 different primers were included in just 18 multiplex PCR tubes. Heteroduplex analysis of double-stranded PCR products and gene scanning of fluorescently labelled single-stranded PCR products are the key factors in this system that avoid false positives. At present, BIOMED-2 multiplex tubes have been widely used in the diagnosis of lymphohematopoietic systems, as well as providing suitable PCR targets for MRD detection. They can detect virtually all malignant clonal proliferations of lymphocytes with high sensitivity and specificity (9). Gabert J et al. published the study with the third highest betweenness centrality related to MRD. The measure of MRD in various types of leukemia has been shown to provide independent prognostic information for treatment stratification. To solve the problem of the lack of standardized diagnostic methods for large-scale MRD studies in multicenter treatment trials, 26 university laboratories from 10 European countries collaborated to establish a standardized protocol for the analysis of major leukemia FGs based on TaqMan RQ-PCR technology. Accurate quantitative measurement of FGs can be applied to 35%-45% of ALL and AML cases and in more than 90% of CML cases. This standardized protocol of RQ-PCR detection and analysis of FGs transcripts is crucial for the molecular determination of MRD and the management of patients in multicenter treatment (36). The above three articles mainly involved the MRD detection of hematological malignancies based on RQ-PCR, focusing on the standardization of RQ-PCR detection procedures and interpretation of results, as well as quality control of detection. They play a landmark role in the development and application of MRD.

**Figure 9:**
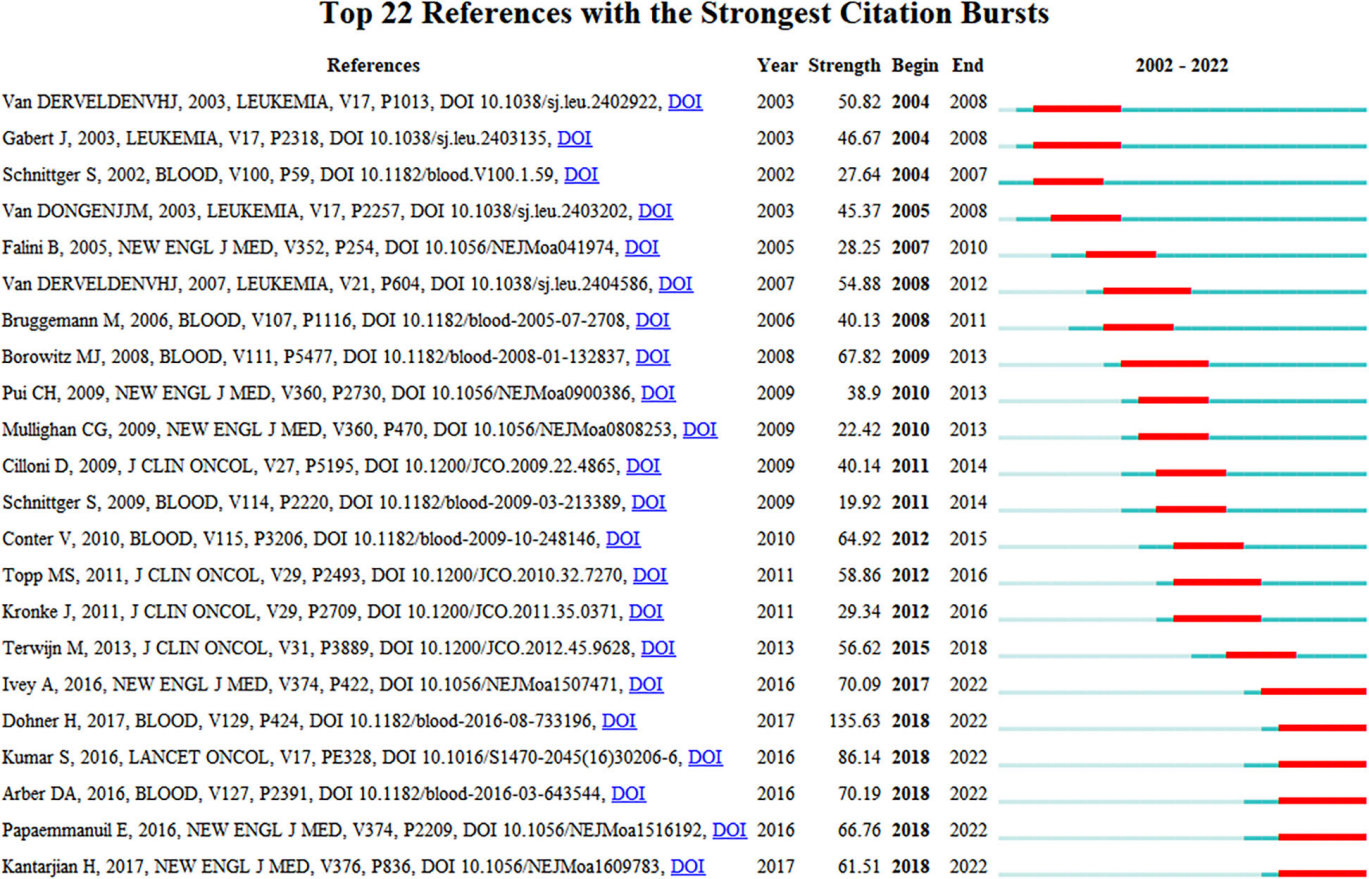
The top 22 references with the strongest citation bursts from 2002 to 2022.

Citation burstiness refers to the citation frequency of a paper increasing sharply over a period of time. The identification of the 22 most impactful references with the most pronounced citation bursts was accomplished by imposing a minimum burst duration of 4 years, as depicted in **Figure 9**. Among these 22 references, 6 had citation bursts that lasted until 2022, and their burst strengths were all above 60, with the highest strength being 135.63, which was significantly higher than that of the other references. Through the analysis of these six publications, we found that the application of MRD in AML, MM, and ALL is the research frontier in this field. Evidenced by the intensively strong citation burst of co-cited references from 2017 to 2022, there has been a growing interest in MRD-related research in recent years.

## 4 Discussion

### 4.1 Primary outcomes

Based on the information of 8,913 papers on MRD from January 1, 2002, to October 30, 2022, obtained from the WoSCC, this study reviewed the development of this field employing a bibliometric system and visualized the research results through VOSviewer, CiteSpace, RStudio, and a bibliometric online analysis platform. We identified highly productive countries and their collaborative networks, contributions of institutions, core journals in the field, revealed important authors, and explored keywords, co-cited references, etc. We also found research hotspots at different times, and keywords and articles involved in these research hotspots are presented.

### 4.2 Development trend of MRD research

The number of articles can reflect the development trend of a research field over the years. According to the 8,913 published articles related to MRD retrieved by WoSCC, the publication trend in the past 20 years can be divided into three stages. In the first stage (2002-2011), the average number of articles published was 273.7 per year, and the development trend of the field did not change too much. In the second stage (2012-2018), the number of papers published increased year by year, and the academic development trend was good. In the third stage (2018-2022), the output of scientific research showed an explosive trend, and the number of published papers increased by more than 100 per year. It shows that MRD research has seen a steady rise in academic achievement over the past two decades, beginning with the early stages of knowledge accumulation, followed by the middle stages of preparation, and culminating in the current period of vigorous development. In recent years, with the progress of detection techniques (RQ-PCR, MFC, NGF, or NGS) and detection methods (based on different identified targets) (37–40), MRD research has reached a new level. At the same time, driven by some consensus documents and application guidelines (16, 19, 36, 41), MRD monitoring has become more normalized and standardized in many diseases, which makes it more clinically significant. Due to these positive factors, research on MRD has emerged in recent years. This has resulted in a mature research field with a strong knowledge base and abundant practical experience. Overall, the number of publications in the field of MRD research is on the rise, and this research has great prospects and potential in the future.

### 4.3 The situation of countries/regions and institutions in MRD research

In terms of the performance of countries/regions, the United States ranks first in the total number of articles published, which has steadily increased year by year. Articles published by the Netherlands were cited the most frequently, as well as those from Germany, Italy, the United Kingdom, and France, all with an H-index of more than 100. These countries have the most advanced medical institutions, adequate financial resources, and a large number of excellent medical personnel. Scientists from the United States and Europe have been leading the way in research on hematological malignancies for many years. At the same time, MRD, which is significantly related to the recurrence and prognostic stratification of such diseases, has also been deeply studied and well developed. Due to the good scientific atmosphere, the performance of Chinese scientists in the field of MRD research has become increasingly prominent in recent years, and the number of articles published each year after 2019 has ranked second only to the United States, showing that China is paying increasing attention to research in this field and will become a more important participant in the future. Then, we calculated the volume of publications and multicountry collaboration in the countries/regions where the corresponding authors were located. Although China has published more articles than many countries, it still needs to further strengthen international cooperation to promote the better development of MRD research. Among the top 10 contributing institutions in the field of MRD research, nine were from the United States, and one was from China, indicating the US’s strong leadership in the field owing to the significant contributions made by its research institutions. Among all institutions, the University of Texas MD Anderson Cancer Center published the most articles by far and had the greatest impact.

### 4.4 Representative authors and leading journals on MRD research

Through our exploration, a group of representative authors emerged: Kantarjian HM from the University of Texas MD Anderson Cancer Center contributed the most publications, van Dongen JJM from Leiden University Medical Center had the most cited articles, and the research papers of Pui CH of St. Jude Children’s Research Hospital were the most frequently co-cited in the field of MRD. We also found that several representative scholars have formed stable research teams in the field of MRD research, which have made outstanding contributions to the good development of MRD. The popular journals in the research field are also something that researchers are interested in and want to learn about, we can see their information in **Table 3** and **Supplementary Table S1**. A large number of MRD-related articles have been published in *Blood*, *Leukemia*, and the *British Journal of Haematology*, which were the top journals in their categories, with IF of 25.669, 12.897, and 8.615, respectively. However, as a whole, the source journals of the literature cited by the MRD studies seem to have higher IF. This also implies that both the output quality and knowledge source of MRD are of high academic level.

### 4.5 Hotspots and frontiers of MRD research

Keywords are indicative words that are highly related to the research topic, which can indicate or express the characteristics of the topic content of the paper. In this study, the keyword analysis of MRD research in the past 20 years found that the diseases closely related to MRD are mainly AML, MM, ALL, CML, NHL and other hematological malignancies, suggesting that the MRD assessment system is more mature in clinical application in the field of hematological diseases. Because MRD is mainly based on liquid biopsy, which permits direct observation of residual tumor factors or small lesions in patient peripheral blood (PB) or bone marrow (BM) samples, it affords significant advantages in the detection of hematological malignancies. MRD monitoring in ALL, MM, and CML is a well-established standard (19, 42, 43). In AML patients, detection of MRD during complete remission has significant independent prognostic value for relapse and survival (44). The evaluation of MRD is considered useful to more precisely define AML response to intensive chemotherapy, thereby refining risk stratification (45). The establishment of MRD detection and monitoring in patients with solid tumors remains challenging due to the amount of circulating tumor cells, circulating tumor DNA, and exosomes in the PB of patients with solid tumors being very low, and the tumor mutation genes shed into the PB are heterogeneous (46). However, some studies have shown that MRD monitoring is also valuable in solid tumors, such as lung cancer, gastric cancer, and squamous cell carcinoma of the head and neck (47–50).

At the same time, words related to hematological oncology treatment, such as stem cell transplantation, BM transplantation, and chemotherapy, are also closely related to MRD. MRD evaluation after hematopoietic stem cell transplantation is a critical tool for predicting treatment outcomes and guiding the chemotherapy process (51–54). MRD measured before allogeneic hematopoietic stem cell transplantation was closely related to the prognosis of children with ALL. The study found that the presence of MRD identified a risk of relapse that was 9.5-fold higher and a risk of death that was 3.2-fold higher than the risk among patients without MRD, and pretransplant MRD was the only significant factor for relapse and death (7). In one study, serial MRD monitoring was performed on PB and BM samples from 39 patients with mantle cell lymphoma, and intensification therapy after induction therapy significantly improved the molecular remission rate (46% to 74%). Furthermore, the 3-year probability of remaining progression-free after induction chemotherapy was 82% among MRD-negative patients and 48% among MRD-positive patients. MRD detection following induction immunochemotherapy may be used for risk-adapted therapy (6). It is worth noting that “AIEOP-BFM” appears in the high betweenness centrality keywords. AIEOP-BFM has made considerable contributions to pediatric ALL. In pediatric B-cell precursor ALL treated with the AIEOP-BFM ALL protocol, the quantitative measure of MRD is reliable and valuable in predicting the clinical outcome and risk of relapse (55).

In addition, the keywords related to MRD detection techniques included PCR, flow cytometry, and RT-PCR, etc., indicating that these methods are the mainstream techniques for MRD detection. Genetic alterations can be found in cancer patients with normal cytogenetic karyotypes and may be considered suitable as PCR targets for monitoring MRD (56). Moreover, many markers, such as immunoglobulin, T-cell receptor gene rearrangements and FGs, have been well established as potential PCR targets for MRD and are being continuously optimized (9, 57, 58). PCR monitoring of MRD ensures the highest sensitivity and specificity, however, patients with some diseases have no genetic markers suitable for PCR monitoring, and therefore, MFC is a better choice. Cancer cells will show distinct immunophenotypic deviation from normal cells in the body, and this deviation is the precept by which MFC identifies cancer (59). Many studies have shown that MRD measured by MFC is an independent risk prognosticator in AML (60). Furthermore, NGS has emerged as a crucial approach for monitoring MRD in recent years, especially in some special situations, such as AML or myelodysplastic syndrome, and MRD monitoring based on NGS has more prominent advantages (61). MM is characterized by the clonal expansion of monotypic plasma cells in the BM, and residual plasma cells in the BM may be related to the recurrence of the disease. Based on the recommendations of the IMWG consensus, the methods of BM MRD assessment include NGF and next-generation sequencing NGS (19). The MRD detection methods mentioned above have their characteristics and advantages but also have the limitation of a single application. Currently, a combination of these techniques may provide a more effective and comprehensive assessment of clinical treatment (62).

## 5 Strength and limitations

Bibliometrics is a comprehensive knowledge system that integrates mathematics, statistics, and philology and pays attention to quantification. Visualized analysis can provide a clear depiction of the development process, research status, and research hotspots of a given field, thereby serving as a reference for further research. However, due to some objective factors, there are certain limitations in this research. First, the samples in this study are only from the Science Citation Index Expanded database in the WoSCC. Although the WoS covers a wide range of journals and is a mainstream source of data in the bibliometrics field, there may still be several articles on this topic that are not counted by WoS. Nevertheless, it is worth noting that WoS is the most frequently employed database in bibliometric studies (63, 64). Second, to ensure the integrity of information in the analyzed literature, the data collected in this study were limited to articles and review articles, and literature types such as meeting reports, books, and case reports were not included. Third, although the software used for bibliometric analysis and the data collected are objective, the subjective nature of the analysis and interpretation cannot be avoided. Finally, recent high-quality studies may not be as widely cited due to their recentness, so influential studies may need to be highlighted by several years of high citation.

## 6 Conclusion

This study used data information combined with visualization software such as VOSviewer, CiteSpace, RStudio software package, and an online analysis platform of bibliometrics to conduct a bibliometric analysis of MRD-related publications and explore the global trend, knowledge framework, research hotspots, and frontiers in this field over the past two decades. With the continuous development of detection technologies such as PCR, MFC, and NGS and the ability to identify the genetic lesions involved in human malignancies, research based on MRD is also becoming more in depth. With the efforts of many scholars, some recognized MRD evaluation criteria and diagnostic consensus have been established in malignant hematological diseases such as AML, MM, and ALL, which have guiding significance for determining the level of radiotherapy and chemotherapy that a patient should receive, as well as the prognosis stratification and recurrence risk evaluation of patients. The findings of this study can improve the understanding of researchers and scholars in the field of MRD and help them to identify new ideas and research directions, which will further promote the development of MRD research. In addition, for scholars who are new to the field or interested readers, this paper provides them with a complete perspective on the development and evolution of MRD, allowing them to quickly find information on representative scholars, important research institutions, and landmark references in the field.

## Data availability statement

The original contributions presented in the study are included in the article/**Supplementary Material**. Further inquiries can be directed to the corresponding authors.

## Author contributions

ZY and LX designed the research subject and interpreted results, and independently searched, downloaded, and analyzed the data. LX analyzed the data and wrote the manuscript. JZ searched the literature and screened all potentially eligible studies. HL reviewed and corrected the manuscript. TN critically revised the manuscript. All authors contributed to the article and approved the submitted version.

## Supplementary material

Supplementary Figure 1 Search strategy for this bibliometric research.

Supplementary Figure 2
**(A)** The co-occurrence network map of the primary authors in the MRD field. **(B)** The co-occurrence network map of co-cited authors in the MRD field.
