# Supplementary figures and images for: The evolution of minimal residual disease: key insights based on a bibliometric visualization analysis from 2002 to 2022
Source: Front Oncol. 2023 Jul 18;13:1186198. doi: 10.3389/fonc.2023.1186198 (PMC10391156; doi:10.3389/fonc.2023.1186198)

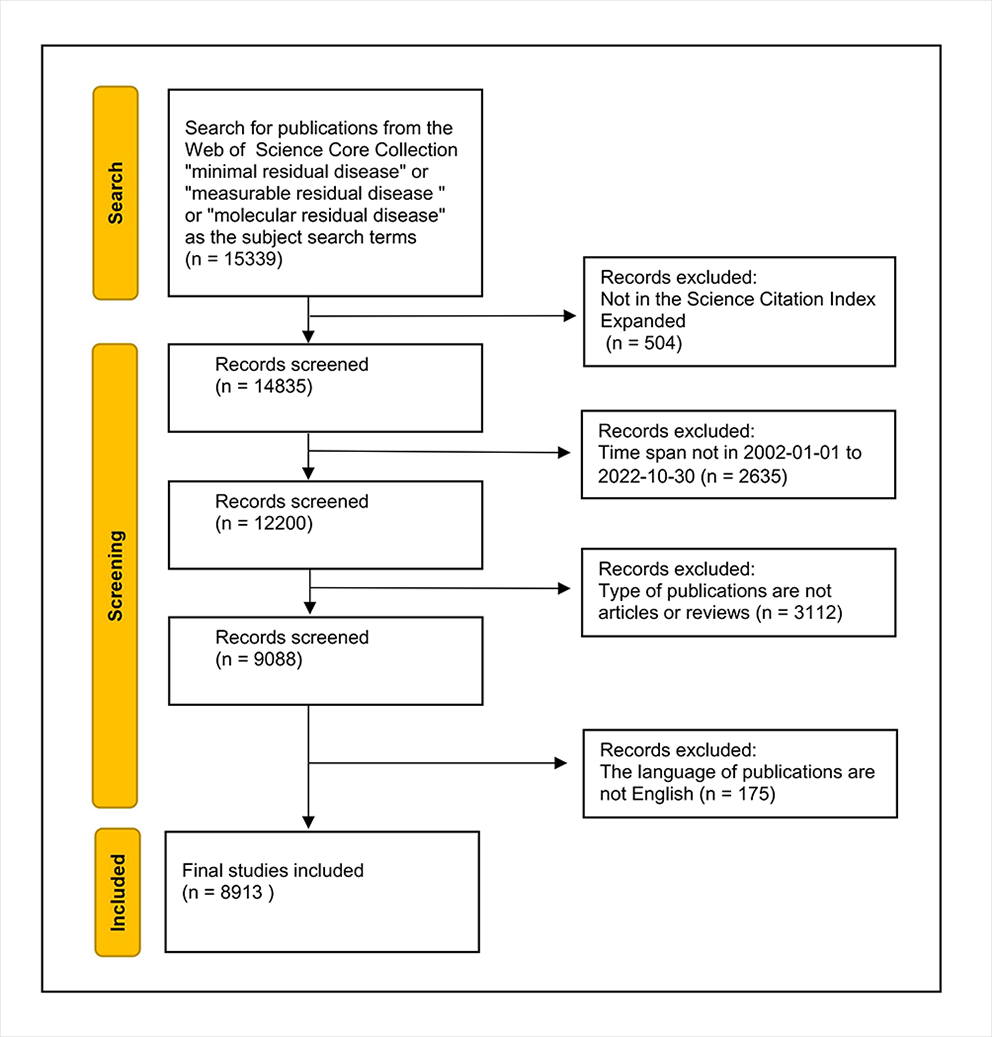

Supplement: Supplementary Figure 1 — Search strategy for this bibliometric research. [file Image_1.tif]

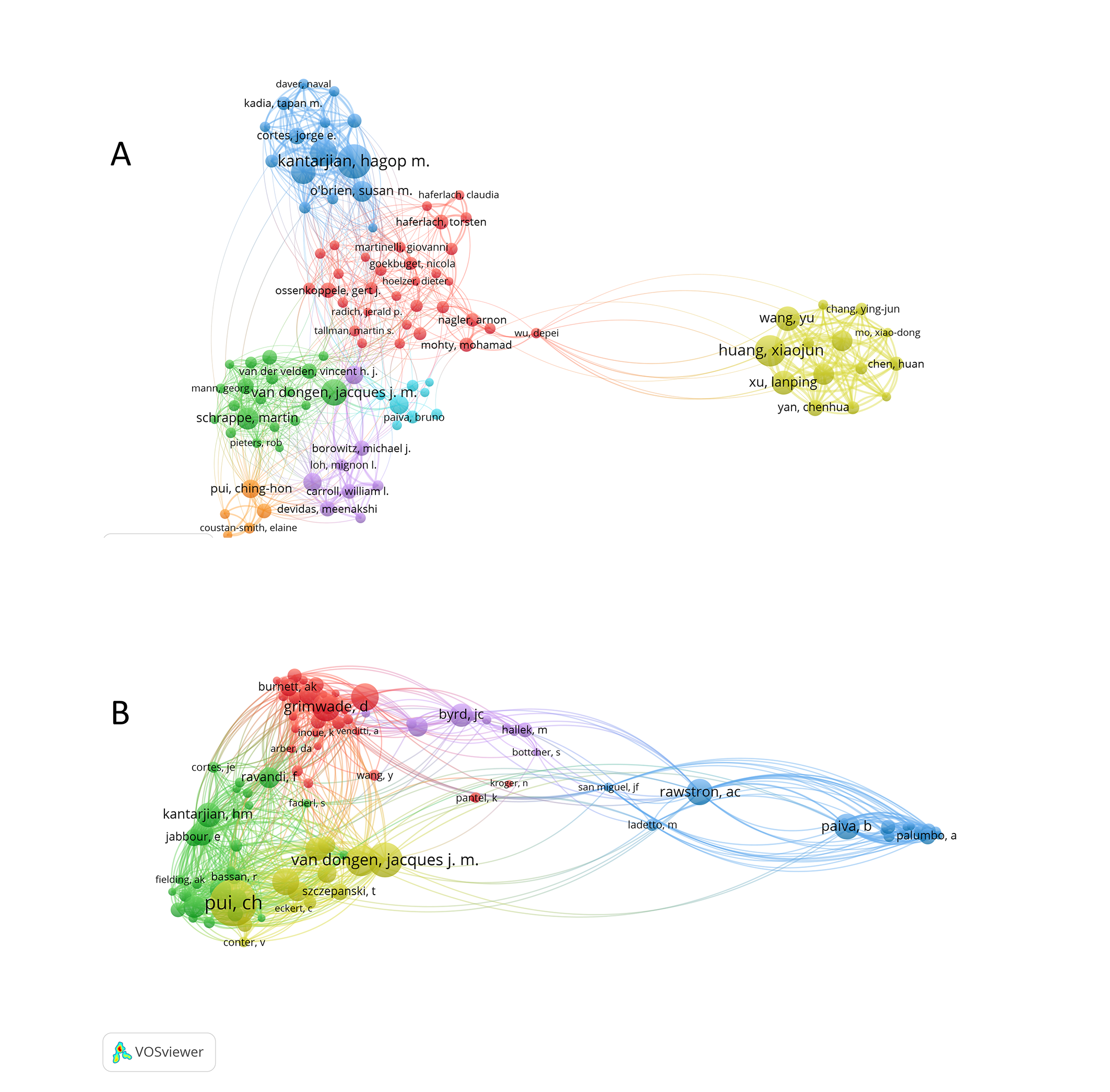

Supplement: Supplementary Figure 2 — (A) The co-occurrence network map of the primary authors in the MRD field. (B) The co-occurrence network map of co-cited authors in the MRD field. [file Image_2.tif]
